# Class switch towards spike protein-specific IgG4 antibodies after SARS-CoV-2 mRNA vaccination depends on prior infection history

**DOI:** 10.1038/s41598-023-40103-x

**Published:** 2023-08-13

**Authors:** Petra Kiszel, Pál Sík, János Miklós, Erika Kajdácsi, György Sinkovits, László Cervenak, Zoltán Prohászka

**Affiliations:** 1 Research Group for Immunology and Hematology, Semmelweis University-Eötvös Loránd Research Network (Office for Supported Research Groups), Budapest, 1085 Hungary; 2 Department of Internal Medicine and Hematology, Semmelweis University, Budapest, 1088 Hungary

**Keywords:** Immunology, Disease prevention

## Abstract

Vaccinations against SARS-CoV-2 reduce the risk of developing serious COVID-19 disease. Monitoring spike-specific IgG subclass levels after vaccinations may provide additional information on SARS-CoV-2 specific humoral immune response. Here, we examined the presence and levels of spike-specific IgG antibody subclasses in health-care coworkers vaccinated with vector- (Sputnik, AstraZeneca) or mRNA-based (Pfizer-BioNTech, Moderna) vaccines against SARS-CoV-2 and in unvaccinated COVID-19 patients. We found that vector-based vaccines elicited lower total spike-specific IgG levels than mRNA vaccines. The pattern of spike-specific IgG subclasses in individuals infected before mRNA vaccinations resembled that of vector-vaccinated subjects or unvaccinated COVID-19 patients. However, the pattern of mRNA-vaccinated individuals without SARS-CoV-2 preinfection showed a markedly different pattern. In addition to IgG1 and IgG3 subclasses presented in all groups, a switch towards distal IgG subclasses (spike-specific IgG4 and IgG2) appeared almost exclusively in individuals who received only mRNA vaccines or were infected after mRNA vaccinations. In these subjects, the magnitude of the spike-specific IgG4 response was comparable to that of the spike-specific IgG1 response. These data suggest that the priming of the immune system either by natural SARS-CoV-2 infection or by vector- or mRNA-based vaccinations has an important impact on the characteristics of the developed specific humoral immunity.

## Introduction

According to clinical trials, vaccination against Severe Acute Respiratory Syndrome Coronavirus 2 (SARS-CoV-2) is the most effective strategy to prevent severe COVID-19 disease^1^. The established vaccination methods are diverse, but all of them result in immunity against the spike protein of SARS-CoV-2. Different vaccines are available in most countries. The most widely applied types of vaccines against SARS-CoV-2 range from inactivated vaccines to non-replicating viral vector vaccines and recently introduced mRNA vaccines^2^. For primary immunizations, mRNA vaccines such as BNT162b2 by Pfizer-BioNTech, (hereafter Pfizer-BioNTech) and mRNA-1273 by Moderna (hereafter Moderna), vector-based vaccines such as ChAdOx-1 by AstraZeneca (hereafter AstraZeneca), Sputnik V by Gamaleya (hereafter Sputnik), Janssen by Johnson&Johnson (hereafter Janssen) and inactivated vaccines such as BBIBP-CorV by Sinopharm (hereafter Sinopharm) were introduced in Hungary, where 61.6% of the total population was fully vaccinated by the end of December 2021^3^.

It is already known that SARS-CoV-2 naïve individuals and previously infected individuals show distinct immune responses to mRNA vaccinations. In naïve individuals, the T cell response is increased after the second dose of the Pfizer-BioNTech vaccine, whereas subjects who recovered from COVID-19 reached peak levels after their first dose^4^. Similar results were found for the spike-specific antibody response; previously infected individuals reached similar specific antibody levels after their first vaccination as non-infected individuals after their second dose of vaccine^5^. After vector-based vaccinations, the level of neutralizing memory B cell-derived antibodies appeared to be significantly lower in Sputnik recipients than in COVID-19 convalescent patients^6^. Besides this, different quantities and quality of CD4+ T cell, CD8+ T cell, and antibody responses were induced by mRNA-based (Moderna and Pfizer-BioNTech), vector-based (Janssen), and protein-based vaccines (Novavax). In this study, mRNA vaccines were proven to be the most immunogenic in terms of the antigen-specific responses, although a decrease in neutralizing antibody titers was demonstrated after 6 months^7^. To maintain high specific antibody levels, booster vaccination was introduced by August 2021, and 32.7% of the whole population in Hungary received at least one dose of a booster vaccine. As booster vaccines, Pfizer-BioNTech, Moderna, AstraZeneca, Janssen, or Sinopharm were applied^3^.

The most abundant immunoglobulin isotype in the human serum is immunoglobulin G (IgG). Its subclasses are highly conserved but differ in their constant regions. Each subclass has a unique profile in terms of antigen binding, immune complex formation, complement activation and triggering of effector cell activation. After antigenic stimuli, IgG3 and IgG1, the two main complement-activating subclasses are secreted first, whereas IgG2 and IgG4, which are formed later, may play a role in attenuating inflammation due to their inability to activate complement^8^. It is known that antibody responses to viral protein antigens are mainly restricted to IgG1 and IgG3^9,10^. IgG2 is stimulated primarily by carbohydrate antigens, whereas IgG4 is produced in response to helminthic infections or to prolonged antigen stimulations^8^. However, IgG subclasses produced against protein antigens depend on factors other than the type of pathogens, such as T-helper cell response, and the route and the site of infections^11^.

In immunological research on SARS-CoV-2, the relationship between specific IgG titer and memory recall response has not been fully investigated^12,13^. Data on single-cell sequencing and flow cytometry have recently shown substantial IgG4-switched, spike-binding memory B cell populations following mRNA vaccination^14^. Another study revealed that adenovirus-based vaccines did not elicit a long-term spike-specific IgG4 response^15^. Further studies are needed to characterize antibody isotypes and subclasses generated in response to different vaccinations and natural SARS-CoV-2 infections.

## Objectives

In the present study, we investigated whether the SARS-CoV-2 infection and the vaccination (with different types of vaccines) induced similar spike-specific IgG subclass pattern and whether these patterns were influenced by the chronological order of natural virus infection and vaccination or not. We also aimed to monitor the concentration of each spike-specific IgG subclass in a period of three to four months to estimate the stability of the antiviral humoral immune response. Comparing the levels of spike-specific IgG subclasses after vaccination and infection may provide insight into the mechanisms that drive the antibody response in both conditions.

## Results

### Vaccinated and COVID-19 cohorts

**Figure 1:**
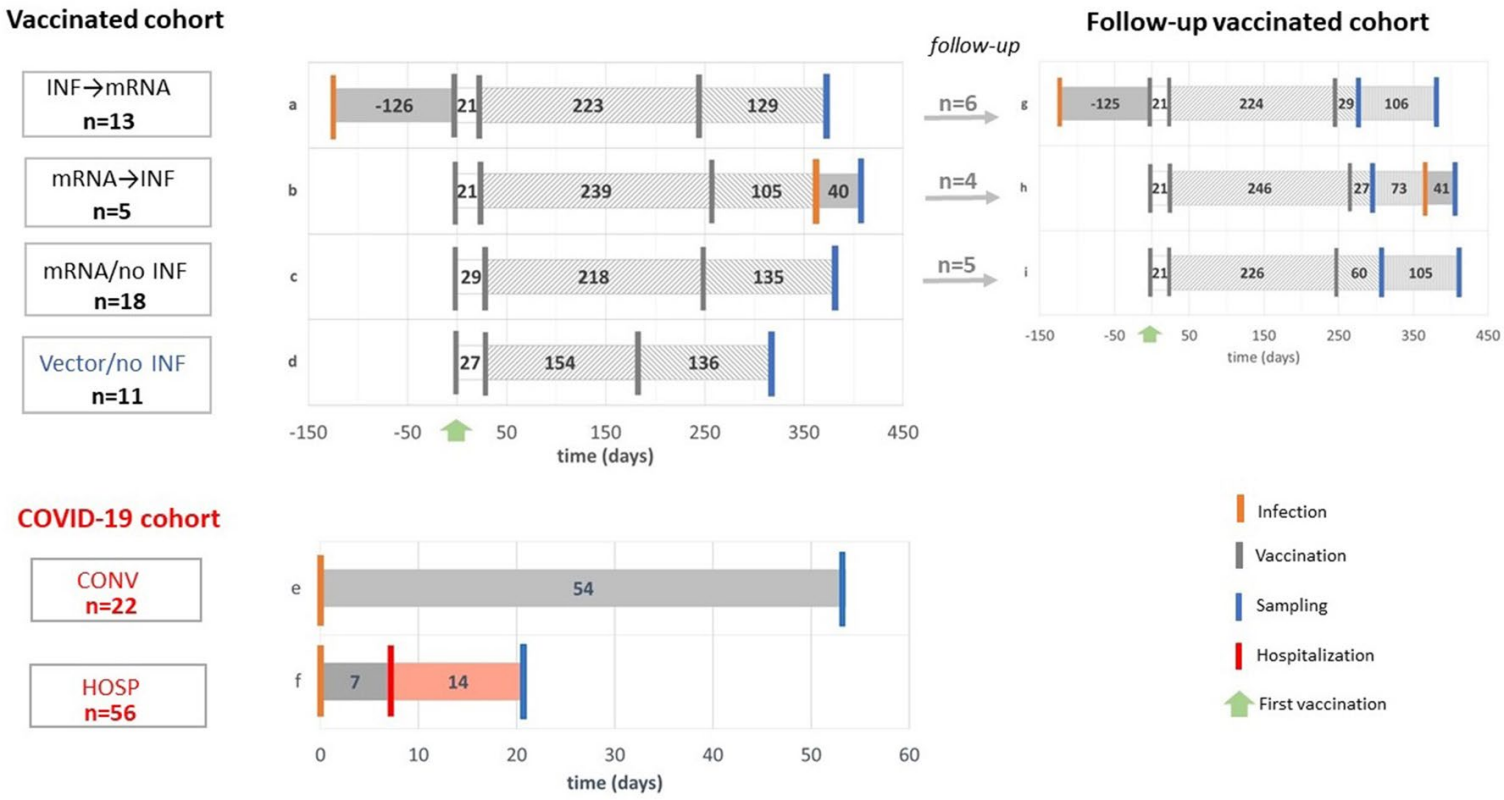
Scheme of the study, study groups. Forty-seven volunteers were included in the vaccinated cohort, as detailed in Table 1. Serum samples from the **INF → mRNA** (**a**), **mRNA → INF** (**b**), **mRNA/no INF** (**c**) and **Vector/no INF** (**d**) groups were collected on median day 135 after their booster vaccinations. The COVID-19 cohorts included 22 convalescent donors (**CONV**, **e**) and 56 hospitalized COVID-19 patients (**HOSP**, **f**) based on positive anti-SARS-CoV-2 antibody levels. Their samples were collected after the infections on median days 54 and 21, respectively. Furthermore, 15 individuals from the **INF → mRNA** (**g**), the **mRNA → INF** (**h**), and **mRNA/no INF** (**i**) groups could be followed up after their booster vaccinations on median days of 37 and 160. Schematic representation of infection, vaccination, sampling and hospitalization time points are shown in orange, grey, blue and red lines, respectively. Green arrows indicate the time of the first vaccination. Numbers indicate group medians (days).

We recruited 47 healthy volunteers for the vaccinated cohort in Budapest, Hungary. Of these, 36 subjects (median age = 41 IQR [32–51]); 64% female) were vaccinated with mRNA vaccines (Pfizer-BioNTech or Moderna) and 11 (median age = 51 IQR [43–57]); 45% female) with vector-based vaccines (Sputnik or AstraZeneca). Serum samples were collected on median day 128 IQR [100–157] after the booster vaccination. The COVID-19 cohort included serum samples from convalescent participants (median age = 45 IQR [34–55]); 41% female) and hospitalized patients (median age = 69 IQR [58–78]); 39% female) taken on median days 54 IQR [45–64] and 21 IQR [9–41] after infection, respectively. (Fig. 1, Table1).

**Table 1:** Demographic data of vaccinated and COVID-19 cohorts.

	INF → mRNA (n = 13)	mRNA → INF (n = 5)	mRNA/no INF (n = 18)	Vector/no INF (n = 11)	CONV (n = 22)	HOSP (n = 56)
Age in years, median [IQR]	42 [34–51]	42 [38–52]	35 [32–60]	51 [43–57]	45 [34–55]	69 [58–78]
Male, n (%)	5 (38)	2 (40)	6 (33)	6 (55)	13 (59)	34 (61)
Female, n (%)	8 (62)	3 (60)	12 (67)	5 (45)	9 (41)	22 (39)

Data are expressed as median [IQR] and n (%).

Furthermore, we longitudinally monitored 15 mRNA-vaccinated volunteers at two time points following their booster vaccination on median days 37 IQR [20–60] and 160 IQR [125–173] (Fig. 1). Demographic characteristics of the cohorts are further described in more detail in Tables 1 and 2.

**Table 2:** Characteristics of followed individuals.

	INF → mRNA (n = 6)	mRNA → INF (n = 4)	mRNA/no INF (n = 5)
Age in years, median (IQR)	41 (33–52)	47 (41–52)	34 (29–74)
Male, n (%)	1 (17)	2 (50)	1 (20.0)
Female, n (%)	5 (83)	2 (50)	4 (80.0)

Fifteen volunteers were recruited for the study. Data are expressed as median (IQR) or n (%).

### Levels of the total spike-specific IgG and the total serum IgG in the vaccinated cohort after primary and booster vaccinations and in the COVID-19 cohort

**Figure 2:**
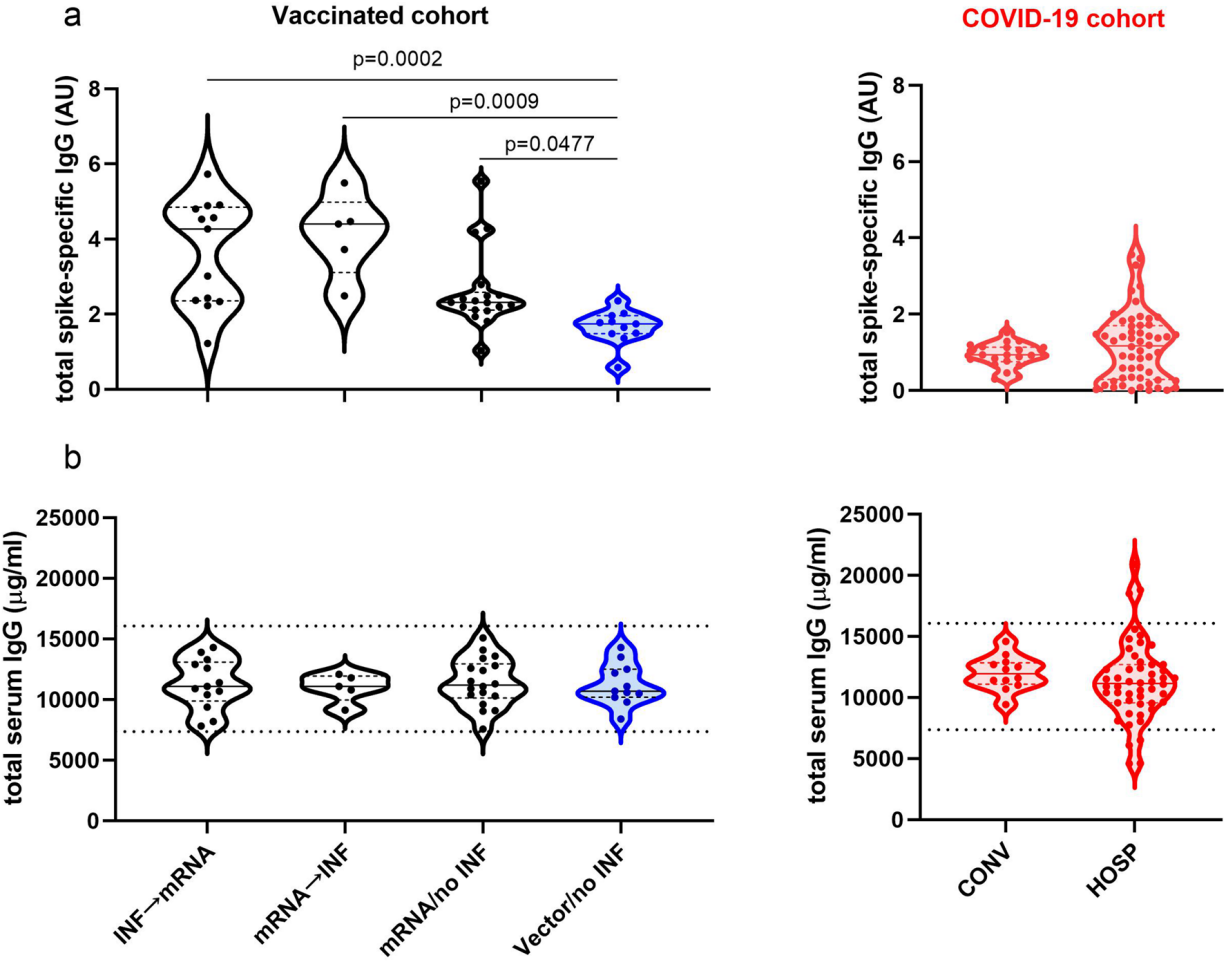
The total spike-specific IgG and the total serum IgG levels in vaccinated and COVID-19 cohorts. Total spike-specific IgG levels were quantified by in-house ELISA, normalized to positive and negative serum samples (**a**). Total serum IgG levels were measured by nephelometry (**b**). Black and blue circles indicate mRNA and vector-vaccinated volunteers in the vaccinated cohort, while red circles represent the COVID-19 cohort. The horizontal solid lines indicate group medians, and the horizontal dashed lines indicate 25–75% percentiles. The dotted lines represent the reference ranges of total serum IgG by Siemens Healthcare Diagnostics Inc. The Kruskal–Wallis test followed by Dunn’s multiple comparisons test is presented for the vaccinated cohort. p < 0.05 was considered statistically significant.

First, total spike-specific IgG and total serum IgG antibodies were quantified in our vaccinated and COVID-19 cohorts. mRNA-based vaccinations resulted in higher total spike-specific IgG levels than in uninfected vector-based vaccinations (Kruskal–Wallis ANOVA, p < 0.0001). Even though the differences between the **mRNA/no INF** group and the infected mRNA group (**INF → mRNA**, **mRNA → INF**) were not significant, there is a trend for higher total spike-specific IgG levels in the infected mRNA groups. The lack of significance is maybe due to the small sample sizes. Similarly to **Vector/no INF** group, we detected low total spike-specific IgG levels in the COVID-19 cohort. Total serum IgG levels were in the physiological ranges and did not show any significant differences among the groups tested (Fig. 2).

### Subclass distributions of spike-specific and total serum IgG antibodies in the vaccinated cohort following primary and booster vaccinations and in the COVID-19 cohort

**Figure 3:**
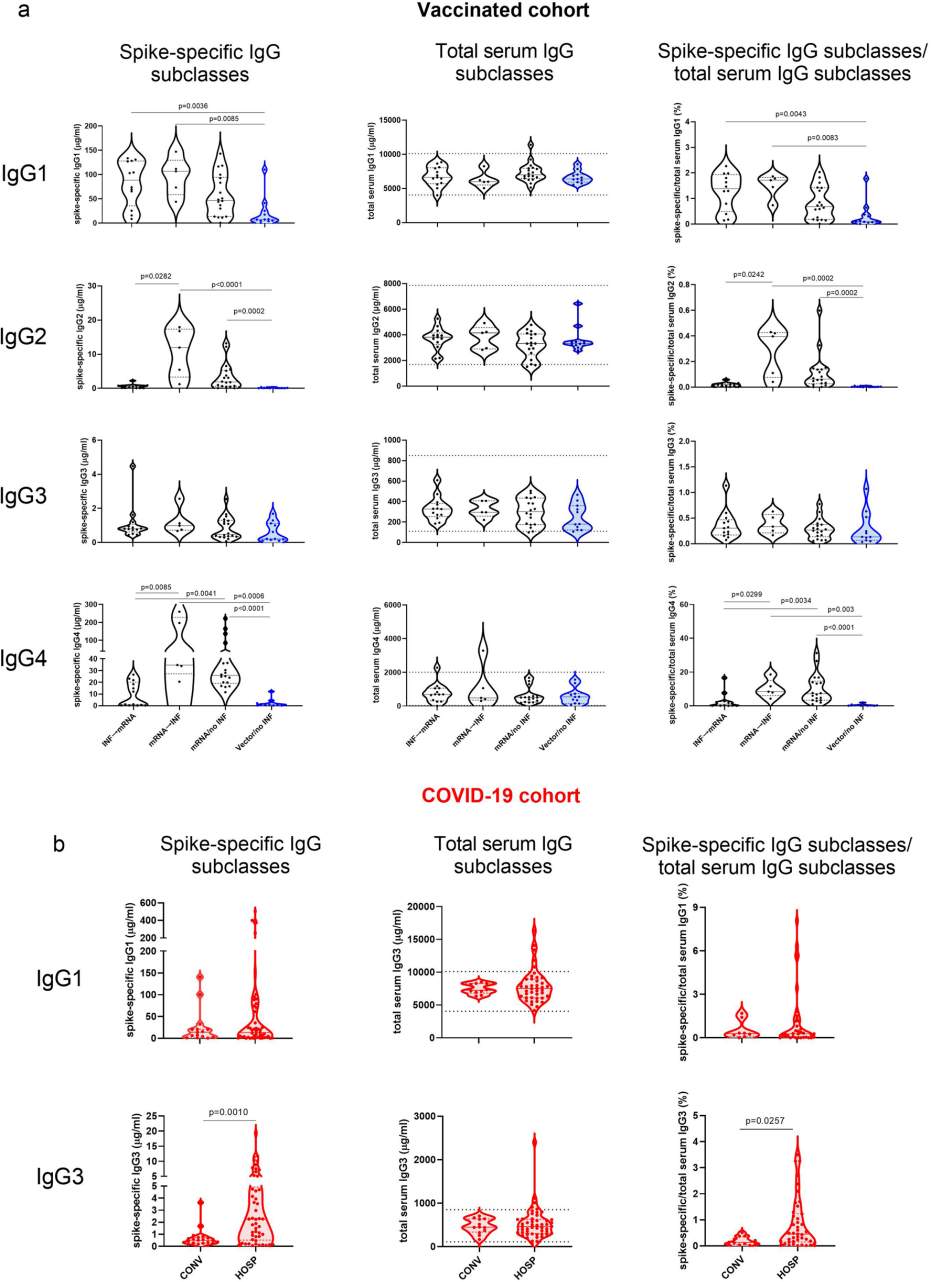
The spike-specific, the total serum IgG subclasses and the proportions of each spike-specific IgG subclass to their total serum IgG antibody subclass in the vaccinated (**a**) and the COVID-19 (**b**) cohorts. Levels of spike-specific IgG subclasses were quantified by in-house ELISA calibrated with purified human IgG 1, 2, 3 and 4 antibodies. Total serum IgG subclass concentrations were measured by nephelometry. Black and blue circles indicate mRNA and vector-vaccinated volunteers in the vaccinated cohort (**a**), while red circles represent the COVID-19 cohort (**b**). The horizontal solid lines indicate group medians, and the horizontal dashed lines indicate 25–75% percentiles. The dotted lines represent the reference ranges of total serum IgG subclass levels by Siemens Healthcare Diagnostics Inc. The values of the Kruskal–Wallis test followed by Dunn’s multiple comparisons test are presented in the vaccinated cohort. p < 0.05 was considered statistically significant.

Individuals both infected and vaccinated with mRNA primary vaccines (**mRNA → INF**, **INF → mRNA**) had high levels of spike-specific IgG1, irrespectively of the time of COVID-19 infection. Their spike-specific IgG1 levels were higher than those of **Vector/no INF** individuals, and similar trends were observed in comparison to the **mRNA/no INF** group. Although the spike-specific IgG3 subclass was one of the dominant specific IgG subclasses in COVID-19 patients, we did not find any substantial differences in spike-specific IgG3 levels measured in our vaccinated groups. Interestingly, high spike-specific IgG2 and IgG4 levels were measured in subjects who received mRNA vaccines first (**mRNA → INF**, **mRNA/no INF**), irrespective of whether they were infected after their vaccinations or not. Moreover, spike-specific IgG4 was a major contribution to the total IgG4 levels. Their proportions were around 10% in the **mRNA → INF** and **mRNA/no INF** groups, far exceeding the ratios that we detected in other subclasses. All of the total serum IgG subclasses were measured in the vaccinated cohort. In the COVID-19 cohort, spike-specific IgG2 and IgG4 subclasses were not detected, therefore we only measured the total serum IgG1 and IgG3 subclasses in this cohort. Notably, we did not find any significant differences among total serum IgG antibody subclass levels (Fig. 3).

### Heatmaps of spike-specific and total serum IgG antibody subclass levels in the follow-up vaccinated cohort

**Figure 4:**
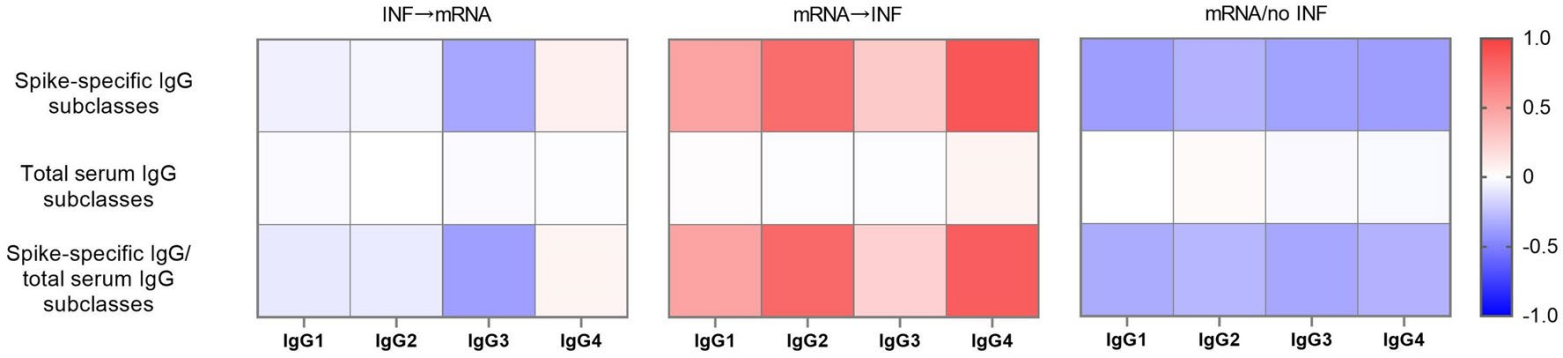
Heatmaps representing the log mean fold change values of spike-specific and total serum IgG subclasses in three follow-up mRNA vaccinated groups between two sampling time points after booster vaccination. The fold changes of spike-specific and total serum IgG subclasses and the proportions of each spike-specific to total serum IgG levels were visualized in heatmaps by the logarithmic values of mean ratios between the second and the first sampling time points. Decreasing trends are shown between 0 and (− 1) and increasing trends were plotted between 0 and 1. The constant level (no change) is indicated as 0.

The heatmap visualizations were performed by the logarithmic values of mean fold change in spike-specific and total serum IgG subclass levels between the two sampling time points after booster vaccination. Subjects in the **INF → mRNA** group showed decreased levels of spike-specific IgG3 and constant levels of spike-specific IgG1, IgG2, and IgG4. Individuals in the **mRNA → INF** group had increasing spike-specific IgG1, IgG2, and IgG4 levels. The concentration of spike-specific IgG3 slightly elevated between the two sampling time points, however, its level was as low as around 1 µg/ml. Levels of spike-specific IgG1 and IgG3 decreased in individuals in the **mRNA/no INF** group. Furthermore, decreasing trends were detected in the antibody subclasses of spike-specific IgG2 and IgG4. Each serum IgG antibody subclass showed a constant level in all vaccinated groups over a period of three to four months. As the concentration of each total serum IgG subclass remained stable, the proportion of the level of each spike-specific IgG subclass to that of the total serum IgG subclass varied similarly to the spike-specific IgG subclasses during the follow-up period. This demonstrates that mRNA vaccinations independently of SARS-CoV-2 infections did not modify the distribution of total serum IgG subclass (Fig. 4). The spike-specific and total serum IgG antibody subclass levels are shown in the follow-up mRNA vaccinated groups between the two sampling time points (Fig. S4).

### Levels of spike-specific IgG subclasses expressed as percentages of all spike-specific IgG antibodies

**Figure 5:**
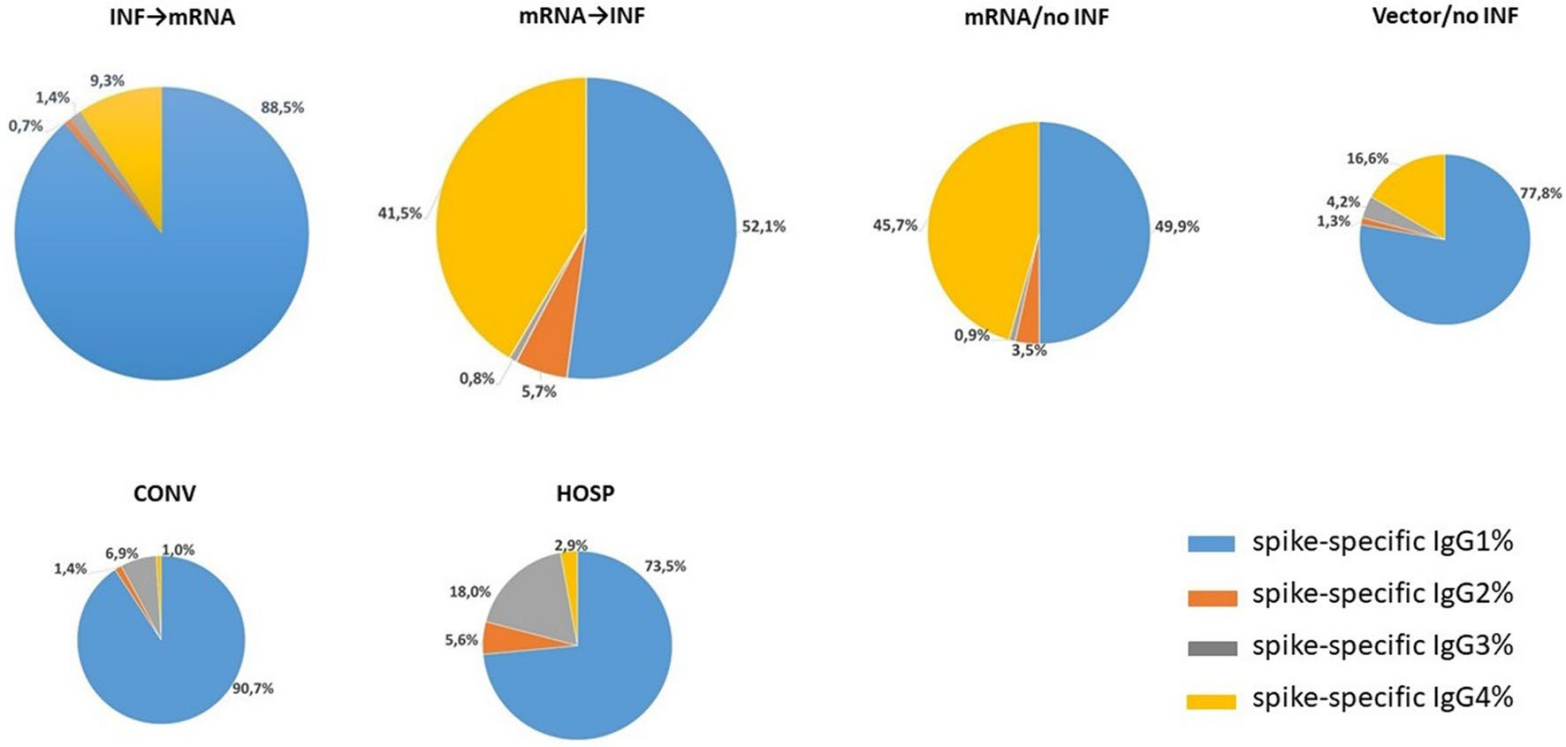
Contribution of each spike-specific IgG subclass to the sum of all spike-specific IgG antibody levels. The percentages of each spike-specific IgG subclass relative to the sum of spike-specific IgG levels are shown for the vaccinated and COVID-19 cohorts. The numbers represent the mean percentages of individuals calculated by dividing each spike specific IgG levels with the sum of all spike specific IgG levels. Schematic representation of spike-specific IgG1%, IgG2%, IgG3% and IgG4% are shown in blue, orange, grey and yellow sectors, respectively. The size of the pie charts indicates the levels of all spike-specific IgG antibodies from Fig. 2.

The percentages of spike-specific IgG4 were higher in the vaccinated groups than in the COVID-19 cohort groups. The proportions of the spike-specific IgG4 subclass to the sum of all spike-specific IgG antibodies were between 1 and 3% in the **CONV** and **HOSP** groups, respectively. In the vaccinated groups, we detected 16.6% of spike-specific IgG4 in the **Vector/no INF** group, whereas its values were as high as 41.5% and 45.7% in the **mRNA → INF** and **mRNA/no INF** groups, respectively (Fig. 5).

## Discussion

In recent years, several different vaccine platforms have been introduced to control COVID-19 disease^2,7^. However, their urgent introduction failed to facilitate the characterization of their mechanism of action^16^.

Here, we had a unique opportunity to compare the humoral immune responses induced by DNA-based non-replicating adenoviral (Ad) vector- (AstraZeneca, Sputnik) and mRNA-based (Pfizer-BioNTech and Moderna) vaccine platforms. In the present study, we determined the levels of total spike-specific IgG and total serum IgG in our vaccinated and COVID-19 cohorts. Individuals vaccinated with vector-based vaccines had lower total spike-specific IgG levels than subjects with mRNA vaccines approximately four to five months after their booster vaccination. Hoogen et al. also showed higher spike S1-specific antibody levels following vaccination with mRNA-based vaccines than vector-based vaccines in the general population in the Netherlands^17^. Furthermore, low total spike-specific antibody levels were detected in our convalescent and acute patient groups of the COVID-19 cohort. Similar results were reported that serum levels of SARS-CoV-2 spike-specific IgG were 5.6 times higher in vaccinated subjects than in infected patients^18^. The measured total serum IgG levels corresponded to normal serum levels, and we did not find any significant differences among our investigated groups^19^. The pattern of spike-specific IgG subclasses in individuals who were infected before mRNA vaccinations was similar to that of subjects who received vector-based vaccines, or COVID-19 patients who did not receive any vaccines. In our COVID-19 patients, the spike-specific IgG1 and IgG3 antibody subclasses were dominantly present on median 21 days post-infection. Several reports also showed that the dominant subclasses in COVID-19 patients were spike-specific IgG1 and IgG3^20^. Here, the predominance of IgG3 in SARS-CoV-2 infection may be explained by the fact that generally, IgG3 appears early in viral infections because it is encoded in the most upstream (5ʹ) constant (C) γ region of the immunoglobulin heavy chain gene locus on chromosome 14 and elicits classical complement pathway activation. The further class switch recombinations follow a one-way direction IgG3 → IgG1 → IgG2 → IgG4 subclasses^21,22^. We found that the pattern of spike-specific IgG subclasses was markedly different in mRNA-vaccinated individuals with or without prior SARS-CoV-2 infection. A further switch towards the distal IgG subclasses (spike-specific IgG4 and moderate spike-specific IgG2) occurred almost exclusively in individuals who had received only mRNA vaccines or who had SARS-CoV-2 infection after mRNA vaccination. Interestingly, the individuals, who experienced a breakthrough infection later than 3 months after the booster vaccination, a marked elevation in spike-specific IgG2 and IgG4 antibody titers were observed at 40 days after their experienced breakthrough infection. Additionally, we were also able to demonstrate that individuals who were infected with SARS-CoV-2 before basic mRNA vaccinations had lower and constant spike-specific IgG2 and IgG4 levels compared to other mRNA vaccination groups at time point later than 4 months after their booster vaccination. Similar to our results, Irrgang et al. also showed that the highest spike-specific IgG4 levels were detected in individuals who experienced a breakthrough infection that occurred later than 3 months after their second immunization with mRNA vaccines^14^. Buhre et al. also confirmed that repeated immunization of naïve individuals with mRNA vaccines increased the proportion of spike-specific IgG4 subclass over time^15^. Moreover, we detected low spike-specific IgG4 concentrations in individuals who were infected before mRNA vaccination. These results are consistent with the findings of Buhre et al. that mRNA primary immunization with preinfection induced little or no long-term spike-specific IgG4 responses. Notably, our group vaccinated with vector-based AstraZeneca and Sputnik vaccines did not show long-term spike-specific IgG2 and IgG4 responses. Similar findings were also demonstrated in the above-mentioned two independent studies^14,15^. The longevity of protective humoral immunity is mainly dependent on the generation of memory B cells and long-lived plasma cells (LLPC)^23^. The different cytokine milieu and interactions between B and T cells may contribute to the emergence of different isotype-switched memory B cells or LLPCs^24^. For instance, different cytokine profiles were described after different types of *Bordetella pertussis* vaccinations. The whole-cell pertussis vaccinations were characterized by an elevated production of T-helper cell type 1 cytokines, such as IFNγ or IL-2. The acellular pertussis vaccines induced a mixed cytokine profile showed elevated IFN-γ production (associated with IL-2), low IL-4, and more abundant IL-5 production^25^. The vaccine composition might also play a role in the induction of specific IgG4 subclass. This mechanism is not yet clearly understood, but the persistent GC response induced by SARS-CoV-2 mRNA-based vaccines might be a feasible explanation in the generation of long-lived bone marrow plasma cells with IgG4 subclass ^14,26^.

In all vaccination groups, subjects developed spike-specific IgG1 and IgG3 antibodies, independently from their prior infection history. Notably, in our vaccinated cohort, we observed higher levels of spike-specific IgG1 in healthy volunteers who were infected before or after their mRNA vaccination than in those vaccinated with vector-vaccines. Consistent with the short half-life of IgG3, spike-specific IgG3 levels were low in the vaccinated groups and did not differ in healthy individuals.

A further aim of our study was to monitor the changes of spike-specific IgG subclasses over a period of 3 to 4 months after the third booster vaccination. Increased spike-specific IgG1, IgG2 and IgG4 responses were observed in the mRNA → INF group, suggesting a typical memory B cell recall response. By contrast, spike-specific IgG1, IgG2 and IgG4 concentrations decreased in the mRNA/no INF group and were fairly constant in the INF → mRNA group. The different behavior of spike-specific IgG3 from that of the other IgG subclasses can be explained by the short half-life of IgG3. Total serum IgG levels did not change during the followed time intervals in the mRNA-vaccinated groups. However, we had some limitations in the first sampling time points after the booster vaccinations. In the mRNA/no INF follow-up group, the first sampling time point was 31 and 33 days later, than in INF → mRNA and mRNA → INF groups, respectively.

More importantly, the percentages of spike-specific IgG4 to the sum of all spike-specific IgG subclasses after mRNA vaccination were similar to the spike-specific IgG1 responses in subjects who were not infected or who were infected after their mRNA vaccination. This supports the phenomenon that pre-existing spike-specific IgG4 memory B cells were reactivated in the mRNA vaccinated groups, where the first encounter was the mRNA vaccine. The mRNA vaccine used for priming immunization seems to be crucial in the emergence of the high IgG4 subclass distribution in the secondary immune response. This phenomenon was also described in another studies^14,15^. Interestingly, similar priming effect was described in children immunized with acellular pertussis or whole-cell pertussis vaccines. Here, in unvaccinated convalescent children the pertussis toxin (PT) -specific IgG1 and IgG3 subclasses were prevalent whereas in children immunized with acellular pertussis vaccines both healthy or breakthrough infected, PT-specific IgG1, IgG2 and IgG4 subclasses were mainly produced^27^.

Our results may indicate that the class switch regulation of spike-specific B cells depends on the type of vaccine (i.e. mRNA versus vector) and the circumstances of the first encounter with the spike antigen (vaccination versus infection). Moreover, this seems to be transmitted at the level of memory B cells, as infection recalls IgG2 and IgG4 besides IgG1 response long after mRNA vaccination.

In contrast to other IgG subclasses, IgG4 has immunosuppressive properties and accounts for only 4–5% of total IgG. Its interaction with the C1q molecule is weaker than that of the other subclasses, therefore, it is unable to activate the classical complement pathway^28^. Because of its low affinity to C1q, but generally high affinity to antigen, IgG4 antibodies act as natural blocking antibodies^29^. Till now, very few studies have reported a vaccine-induced IgG4 responses against infectious diseases. For instance, elevated IgG4 levels were shown in children who had received repeated doses of acellular pertussis vaccinations in contrast with whole-cell pertussis vaccines^30,31^. However, specific IgG4 antibodies have been reported during HIV vaccine development^32^. A recent study has shown that HIV-specific IgG1 and IgG4 subclasses can be used to monitor long-term responses to control virus replication and the eradication of HIV infection^33^. Remarkably, specific IgG4 antibody subclass can appear after natural infections such as measles virus infection^34^.

The conventional adenoviral (Ad) vector is a replication-deficient, non-enveloped double-stranded DNA virus with a transgene expression capacity of up to 7.5 kb^35^. Ad vectors can activate innate immunity, while they are recognized by toll-like receptors (TLRs), such as TLR9, TLR2 and TLR4. It is also known that the recombinant protein-specific IgG3 levels were lower in TLR4-knockout (KO) mice following intravenous injection of Ad vectors. In another study, recombinant protein-specific IgM, IgG2, IgG3 and IgA were also downregulated in TLR2 KO mice^36^. As mRNAs are very large and highly negatively charged molecules, vaccine technology applies a carrier system of lipid nanoparticles (LNP) that facilitates their entry into cells by endocytosis. A major component of LNPs is polyethylene glycolylated lipids, which reduce aggregation and increase the biological half-lives of vaccines. However, several disadvantages have been associated with them, such as increased immunogenecity^37–39^. mRNA vaccines can also stimulate innate immunity by recognizing TLR7 or TLR8 in endosomes to stimulate the production of type-I interferon and proinflammatory cytokines. Besides, the stimulation of intracellular TLR3, TLR7 and TLR9 in B cells can lead to generation of IgG1 and IgG4 positive memory B cells^40^. To reduce the potential harmful effect of mRNA, Karikó et al. replaced uridine with pseudouridine, resulting in mRNA molecules that were not immunogenic and had increased translational capacity^41^. Even more HPLC purification of modified mRNAs resulted in reduced induction of type I interferons and proinflammatory cytokines^42^.

Furthermore, high levels of IgG4 expression can appear after allergen-specific immunotherapy^43^ or in several types of cancers^44^, which is beneficial in allergic diseases but may indicate impaired humoral immunity to tumors. Prior to the COVID-19 pandemic, mRNA technology was mostly developed as a novel cancer therapy. For cancer therapies, such as melanoma, glioblastoma, colorectal cancer, and prostate cancer; the mRNA vaccine candidates are still in clinical trials^45^. Immune memory and the generation of specific class-switched antibodies are mainly dependent on T-cell responses. Robust and persistent follicular helper T-cell (Tfh) response has also been described in lymph nodes of individuals vaccinated by Pfizer-BioNTech vaccine^46^. Further analysis of Tfh cells in lymph nodes may play a crucial role in the effectiveness of mRNA-based vaccinations.

## Conclusions

In summary, our data presented here are a highly relevant confirmation of previous findings that mRNA vaccinations induce high proportion of spike-specific IgG4 antibody responses. In contrast, individuals vaccinated with mRNA vaccines after SARS-CoV-2 infection, as well as naïve individuals vaccinated with vector-based vaccines did not develop significant IgG4 isotyped spike-specific response. Further studies are needed to clarify the relevance of our findings for future mRNA-based vaccine development.

## Materials and methods

### Availability of materials

The antibodies, commercial assays and further reagents applied are summarized in Table 3. Further information on resources and reagents can be obtained from the corresponding author.

**Table 3:** Key resources.

Reagent or resource	Source	Identifier
Antibodies		
Mouse anti-human IgG1Fc-HRP (HP6001)	SouthernBiotech	9054-05
Mouse anti-human IgG2 Fc-HRP (31-7-4)	SouthernBiotech	9060-05
Mouse anti-human IgG3 Hinge-HRP (HP6050)	SouthernBiotech	9210-05
Mouse anti-human IgG4 Fc-HRP (HP6025)	SouthernBiotech	9200-05
Goat anti-human IgG HRP	SouthernBiotech	2040-05
Native human IgG1 protein	abcam	ab90283
Native human IgG2 protein	abcam	ab90284
Native human IgG3 protein	abcam	ab118426
Native human IgG4 protein	abcam	ab90286*
Commercial assays		
GA CoV-2 IgM	Generic assays	REF 3930
GA CoV-2 IgG	Generic assays	REF 3920
Recombinant proteins		
Recombinant SARS-CoV-2 spike His protein, CF	R&D systems, biotecne	10549-CV
Recombinant SARS-CoV-2 spike S1 subunit His-tag protein, CF	R&D systems, biotecne	10569-CV-100
Recombinant SARS-CoV-2 spike S2 subunit His-tag protein, CF	R&D systems, biotecne	10594-CV-100
Recombinant SARS-CoV-2 nucleocapsid His protein, CF	R&D systems, biotecne	10474-CV-050
Software		
GraphPad Prism 9 software	GraphPadSoftwares Inc	https://www.graphpad.com/scientific-software/prism/

*No more available commercially.

### Vaccinated cohort

The whole vaccinated cohort comprised samples from 47 healthy volunteers all of whom were positive for spike-specific IgG antibodies. The **INF → mRNA** (infected before mRNA vaccination) (**a**) and the **mRNA → INF** (infected after mRNA vaccination) (**b**) groups contained 13 and 5 subjects, respectively. The **mRNA/no INF** (not infected mRNA vaccination) group (**c**) included 18 subjects who were not infected at all, whereas the **Vector/no INF** (not infected vector vaccination) group (**d**) included 11 volunteers with vector-based vaccines and no previous infection (Fig. 1). Plasma samples were collected after administration of the 2-dose primary and 1-dose booster vaccines on median day 135 [Interquartile range (IQR) 112–161]. One volunteer from the **INF → mRNA** group received only a 2-dose primary vaccine. Exclusion criteria in the **mRNA/no INF** and **Vector/no INF** groups included the presence of nucleocapsid-specific IgG levels. Nucleocapsid-specific IgG levels in vaccinated cohort are presented in Supplement (Fig. S2).

Individuals in the mRNA vaccinated groups received two primary immunizations with BNT162b2 (Pfizer-BioNTech, New York, NY, USA) or mRNA-1273 (Moderna, Massachusetts, MA, USA) vaccines. The **Vector/no INF** group was immunized with either non-replicating viral vector ChAdOx1 (AstraZeneca, Cambridge, UK) or Sputnik V (rAd26-S/rAd5.S, Gamaleya National Research Center, Moscow, Russia). The third booster vaccines were homologous or heterologous vaccines, such as Pfizer-BioNTech, Moderna, Janssen (Ad26.COV2.S, Johnson&Johnson, New Brunswick, NJ, USA) or inactivated virus BBIBP-CorV (Sinopharm, Covilo, China National Biotec Group, Beijing, China) vaccines.

In the follow-up part of the study, 15 healthy donors who belonged to the **INF → mRNA** (**g**), **mRNA → INF** (**h**) or **mRNA/no INF** (**i**) groups of the vaccine cohort were followed after booster vaccinations on median days 37 [(IQR) 20–60] and 160 [(IQR) 125–173] (Fig. 1). These individuals were immunized with two doses of mRNA vaccines such as Pfizer-BioNTech or Moderna and a third homologous or heterologous (Janssen or Sinopharm) booster vaccine. The infections of individuals who were infected after their mRNA vaccination occurred between the two sampling time points.

### COVID-19 cohort

Our cross-sectional part of the study included 26 outpatients (**CONV**) who volunteered for convalescent plasma donation following SARS-CoV-2 infection and 101 hospitalized COVID-19 patients (**HOSP**) with at least one positive qRT-PCR from a nasopharyngeal swab sample. Disease severity was assessed according to WHO guidelines at the time of sampling. Thirty hospitalized patients did not require oxygen support (WHO category 3), 36 hospitalized patients received nasal oxygen support (WHO category 4), and 35 critically ill patients required intensive care (WHO critical 6, 7 and fatal 8 category). We analyzed 22 subjects (**e**) from the **CONV** group and 56 patients (**f**) from the **HOSP** group. Four subjects and 45 patients were excluded, respectively, because their anti-SARS-CoV-2 IgG levels were not measurable with the commercially available anti-SARS-CoV IgG kit (Generic Assays CoV-2 IgG). The study was approved by the Hungarian Ethical Review Agency (ETT-TUKEB; No. IV/4403-2/2020/EKU). Written informed consent was obtained from the patient or next of kin in accordance with the Declaration of Helsinki. The samples were collected in the first epidemic wave, when the patients were not yet vaccinated against SARS-CoV-2. In our COVID-19 patient cohort, severe acute respiratory syndrome was associated with frequent occurence of diabetes mellitus and malignant diseases (p < 0.0001). Patients, who died later, had four comorbidities, whereas survivors had only two. Increased numbers of in-hospital complications, such as pneumonia, respiratory failure, sepsis, thromboembolic events, and acute kidney injury were more common among those who required ICU treatment or died (p < 0.0001)^47^.

### Measurement of the total spike- and nucleocapsid-specific SARS-CoV-2 IgG antibodies

IgG antibodies against SARS-CoV-2 spike (S) and nucleocapsid (N) proteins were detected using commercially available assays (GA Generic Assays GmbH, Germany). The cut-off (Co) and binding index (BI) were calculated according to the instructions of the manufacturer [Cut-off (Co) = 0.250 + OD_Negative control_, BI = OD sample/Co]. Samples with BI above 0.9 were considered positive.

### Determination of the total spike-specific IgG by in-house ELISA

In-house ELISA tests were performed with SARS-CoV-2 full-length spike (S) recombinant protein (R&D systems, Minneapolis, USA). Briefly, recombinant S protein was coated on 96-well polystyrene microtiter plates (Greiner Bio-One GmbH, Austria) at a concentration of 1 µg/ml in 100 µl of coating bicarbonate buffer (pH 9.8) at 4 °C overnight. After blocking with 1% bovine serum albumin (BSA), we washed the plates thoroughly with phosphate-buffered saline (PBS)-Tween. Patient and control sera were diluted 1:25 and loaded on the plates in duplicates. Plates were further incubated at room temperature for 1 h and developed with HRP-labeled goat anti-human IgG secondary antibody (SouthernBiotech, USA). Absorbance values of samples and positive and negative controls were measured at 450 nm, at a reference wavelength of 620 nm using an automated plate reader (Tecan Group Ltd, Switzerland). Cut-off values were determined by the mean value plus two times the standard deviation (SD) of the negative control.

### Determination of each spike-specific IgG subclass by in-house ELISA

Detailed protocols have been described previously^48^. Briefly, recombinant full-length spike proteins and calibrators of purified human antibodies of IgG1 subclass (from 2000 to 15.6 ng/ml) and IgG2, 3, 4 subclasses (ranging from 1000 to 15.6 ng/ml) (abcam, Cambridge, UK) were coated on 96-well polystyrene plates (Greiner Bio-One GmbH) overnight. After blocking, we added serum samples to the plate in dilutions between 1:25 and 1:250 and incubated at room temperature for 1 h. The bound antibodies were then detected with HRP-conjugated mouse anti-human subclass specific antibodies.

### Determination of total serum IgG and total serum IgG subclasses by nephelometry

The concentrations of total serum (not only spike-specific) IgG and IgG1–4 subclasses were measured by nephelometric assay, using a BN™ II System analyzer (Siemens Healthcare GmbH, Erlangen, Germany). Hereafter, we use the terms total serum IgG and total serum IgG1–4 subclasses. Siemens N Antisera to human IgG, Siemens N AS IgG1/IgG2 and Siemens N Latex IgG3/IgG4 were used according to the instructions of the manufacturer.

The percentage of each spike-specific IgG subclass relative to the sum of all spike-specific IgG levels in the vaccinated and COVID-19 cohorts was determined in the following way. First, the percentages of the IgG subclasses were calculated for each individual by dividing the level of each spike-specific IgG subclass by the sum of the levels of all specific subclasses. Then, the mean percentages of individuals were calculated for each IgG subclass in the respective group.

### Statistical methods

Statistical analyses were performed using GraphPad Prism (GraphPad Software v9.0, San Diego, California, USA). Most variables did not show normal distributions; therefore, the data are presented as medians and interquartile (IQ) ranges. Non-parametric statistical tests, such as Mann–Whitney test for two and Kruskal–Wallis test with Dunn’s post hoc test for multiple independent groups were applied. p values < 0.05 were considered statistically significant.

### Ethics statement

The studies involving human participants were reviewed and approved by the National Scientific and Ethical Committee (ETT-TUKEB; No. IV/4403-2/2020/EKU). Written informed consent to participate in this study was provided by the legal guardian of kin of the participants.

### Supplementary Information

Supplementary Information.

## Data Availability

The raw data supporting the conclusions of this article will be made available by the corresponding author on reasonable request.
