# Supplementary material for: Class switch towards spike protein-specific IgG4 antibodies after SARS-CoV-2 mRNA vaccination depends on prior infection history
Source: Sci Rep. 2023 Aug 13;13:13166. doi: 10.1038/s41598-023-40103-x (PMC10423719; doi:10.1038/s41598-023-40103-x)

Supplementary Information

Class switch towards spike protein-specific IgG4 antibodies after SARS-CoV-2 mRNA vaccination depends on prior infection history

Petra Kiszel*, Pál Sík, János Miklós, Erika Kajdácsi, György Sinkovits, László Cervenak & Zoltán Prohászka

Corresponding author: *Petra Kiszel, E-mail address: kiszel.petra.mta@gmail.com

Supplementary Method

Determination of the total nucleocapsid-specific IgG by in-house ELISA

In-house ELISA tests were performed with SARS-CoV-2 nucleocapsid (N) recombinant protein (R&D systems, Minneapolis, USA). Briefly, recombinant N protein was coated on 96-well polystyrene microtiter plates (Greiner Bio-One GmbH, Austria) at a concentration of 1 µg/ml in 100 µl of coating bicarbonate buffer (pH 9.8) at 4°C overnight. After blocking with 1% bovine serum albumin (BSA), we washed the plates thoroughly with phosphate-buffered saline (PBS)-Tween. Patient and control sera were diluted 1:25 and loaded on the plates in duplicates. Plates were further incubated at room temperature for 1 hour and developed with HRP-labeled goat anti-human IgG secondary antibody (SouthernBiotech, USA). Absorbance values of samples and positive and negative controls were measured at 450 nm, at a reference wavelength of 620 nm using an automated plate reader (Tecan Group Ltd, Switzerland). Cut-off values were determined by the mean value plus two times the standard deviation (SD) of the negative control.

Supplementary Figure S2. The total nucleocapsid-specific IgG levels in vaccinated cohort.

Total nucleocapsid-specific IgG levels were quantified by in-house ELISA, normalized to positive and negative serum samples. Black and blue circles indicate mRNA and vector-vaccinated volunteers in the vaccinated cohort. The horizontal solid lines indicate group medians, and the horizontal dashed lines indicate 25-75% percentiles. Here n.s. indicates not significant (p>0.05).

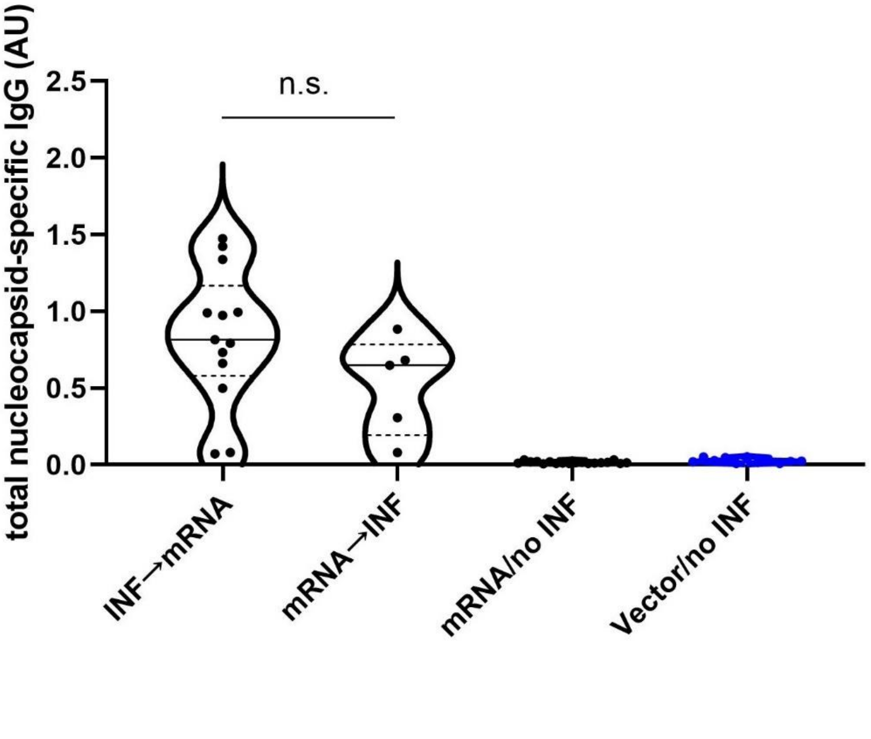

Supplementary Figure S4. Longitudinal monitoring of spike-specific and total serum IgG subclasses in three follow-up mRNA vaccinated groups

The concentrations of each spike-specific IgG subclass were measured by in-house ELISA and levels of each total serum IgG subclass were detected by nephelometry. Furthermore, the proportions of each spike-specific IgG subclass to total serum IgG subclass were also calculated. Each arrow represents data from a single individual.

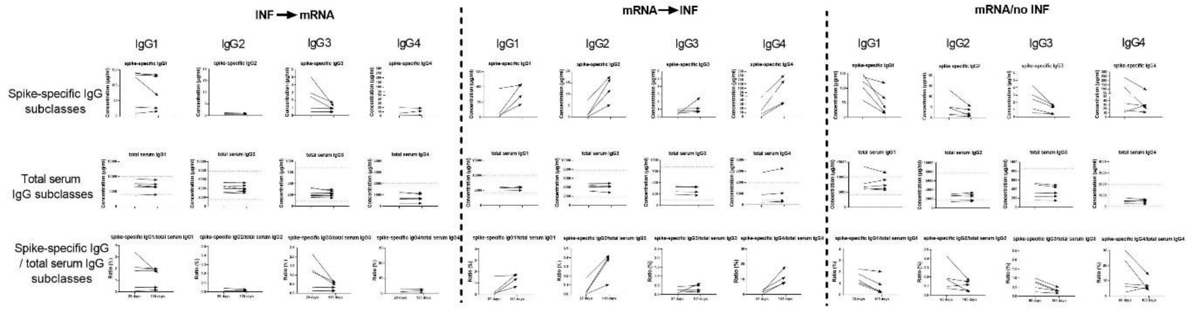

Supplement: Supplementary file 1 — Supplementary Information. [file 41598_2023_40103_MOESM1_ESM.pdf]
